# Differentiating bacterial from viral respiratory tract infections using CRP, SAA, and blood routine parameters: A retrospective cohort study

**DOI:** 10.1016/j.clinsp.2025.100845

**Published:** 2025-11-26

**Authors:** Yu Zhou, Lei Xu, Xiaowu Zhong, Xiaolan Guo, Qiang Ma

**Affiliations:** a Department of Clinical Laboratory, Affiliated Hospital of North Sichuan Medical College, Nanchong, China; b Translational Medicine Research Center of North Sichuan Medical College, Nanchong, China; c School of Laboratory Medicine, North Sichuan Medical College, Nanchong, China

**Keywords:** C-reactive protein, Serum amyloid A, Blood routine parameters, Respiratory tract infection

## Abstract

**Objective:**

Respiratory Tract Infections (RTIs) affect millions globally. This retrospective study evaluates routine hematological parameters for distinguishing bacterial from viral RTIs and develops a novel composite biomarker to enhance diagnostic efficiency, optimize treatment decisions, and improve patient outcomes.

**Methods:**

In the present study, 173 RTI cases (55 bacterial, 118 viral) were enrolled and classified by etiological diagnostic methods (Bacterial culture and identification, or PCR). In addition, 80 healthy controls were also enrolled. Data extraction included SAA, CRP, and blood routine parameters. Biomarkers were assessed individually or in combination using SPSS 26.0 (IBM Corp.) to identify optimal diagnostic models.

**Results:**

Predominant pathogens included Gram-negative bacteria (*Haemophilus influenzae, Klebsiella pneumoniae*) and Gram-positive cocci (*Streptococcus pneumoniae*) for bacterial infections, and influenza A virus/SARS-CoV-2 for viral infections. Bacterial infections showed significantly elevated median levels versus viral infections, such as White Blood Cell (WBC) count is 8.96 vs. 6.22 × 10^9^/L, Neutrophil (Neu) count is 6.69 vs. 4.55 × 10^9^/L, Monocyte (Mon) count is 0.66 vs. 0.49 × 10^9^/L, C-Reactive Protein (CRP) is 68.89 vs. 20.26 mg/L, Serum Amyloid A (SAA) is 280.75 vs. 81.16 mg/L. The performance analysis of differential diagnosis revealed that SAA is AUC = 0.693 (sensitivity 56.4 %, specificity 84.7 %), CRP is AUC = 0.686 (sensitivity 72.7 %, specificity 67.8 %), and Optimal composite (WBC + Neu + Mon + CRP) is AUC = 0.764 (sensitivity 75.2 %, specificity 89.3 %).

**Conclusion:**

The combination of biomarkers (WBC + Neu + Mon + CRP) demonstrates moderate ability (AUC = 0.764) in differentiating bacterial from viral RTIs, which plays a vital role in guiding clinical diagnosis and treatment.

## Highlights

• Bacterial infections exhibit elevated CRP and SAA levels.
• The combined parameter (WBC + Neu + Mon + CRP) achieved an AUC of 0.764.
• The proposed model achieved 75.2 % sensitivity and 89.3 % specificity.

## Introduction

Respiratory Tract Infections (RTIs) rank among the most prevalent clinical diseases worldwide, caused by diverse pathogens including bacteria, viruses, and atypical organisms (*Mycoplasma, Chlamydia*). These infections typically manifest with an acute onset and rapid progression. Bacterial etiologies pose particular concern, as delayed or inappropriate intervention may precipitate severe complications, including bacteremia and sepsis.

Early clinical presentation of RTIs is often nonspecific ‒ characterized by cough, fever, and fatigue ‒ frequently delaying medical attention. Diagnostic challenges are compounded by three critical factors: 1) Symptoms overlap across pathogen types despite divergent treatment requirements; 2) Time delays in etiological diagnostic methods (sputum culture for bacteria; pharyngeal swab for viruses); 3) Technical limitations of conventional methods (low detection rates, prolonged turnaround times, false negatives during low viral-load phases). These constraints perpetuate empirical antibiotic use, exacerbating antimicrobial resistance.^1^ Consequently, it is clinically imperative to rapidly differentiate bacterial or viral respiratory tract infections at admission.

Hematological parameters present distinct diagnostic advantages due to their stability, accessibility, and suitability for continuous monitoring. In the present study, the authors established diagnostic models for differentiating bacterial from viral RTIs using CRP, SAA, and routine blood parameters, which showed that the combination of WBC, Neu, Mon, and CRP (WBC + Neu + Mon + CRP) showed clinically acceptable differentiating capacity.

## Materials and methods

### Objects

#### Ethics

This retrospective study was conducted at Chongqing People's Hospital (April 2023 – March 2024) with approval from the Institutional Review Board (Approval No.: KY S2022-054-01).

#### Participants

A total of 173 patients with RTIs and 80 healthy controls were enrolled.

Inclusion Criteria: 1) Met established diagnostic criteria for RTIs,^2^^,^^3^ presenting with ≥1 clinical feature, such as fever, productive cough, sore throat, fatigue, or abnormal lung imaging; 2) Confirmed etiology by clinical routine diagnostic methods. Bacterial group: qualified sputum samples (gargled/deep-cough specimens; squamous epithelial cells ≤ 10 LPF, WBC ≥ 25 LPF), monomicrobial bacterial culture positive, fungal culture negative. Viral group: bacterial/fungal cultures negative, and single respiratory virus positive.

Exclusion Criteria: Patients were excluded for 1) Non-qualified sputum specimens (risk of colonization/contamination); 2) Any co-infection (fungal, atypical pathogen, bacterial-viral mixed, or polymicrobial); 3) Non-infectious respiratory conditions (acute tonsillitis/laryngitis) or atypical infections (TB, Chlamydia/Mycoplasma); 4) Comorbidities: immunological disorders (e.g., autoimmune diseases), chronic infections (HBV, HCV, syphilis), prolonged/recurrent hospitalization, and age < 18-years.

#### Control group

Eighty healthy subjects were enrolled as the control group during the same period.

#### Data collection

**Fig. 1:**
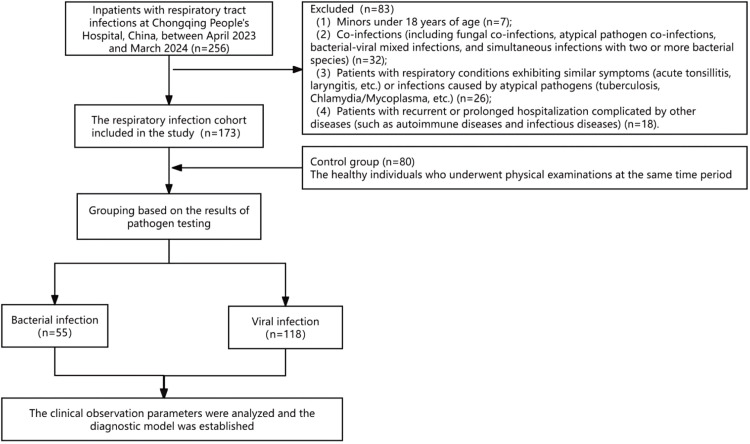
The flow diagram of the present study.

Clinical characteristics and laboratory parameters (CBC, CRP, SAA) were extracted from medical records. The study flowchart appears in Fig. 1, and baseline characteristics are summarized in Table S1.

### Methods for examination of respiratory etiology

1) Bacterial culture and identification: Fresh sputum samples were collected from enrolled patients within 24 h of admission. Specimens meeting quality criteria (≥ 25 polymorphonuclear leukocytes and ≤ 10 epithelial cells per low-power field on Gram staining) were inoculated onto culture plates and incubated at 37 °C with 5 % CO_2_. Bacterial identification was performed using automated systems (Zybio EX2600 and VITEK 2 Compact).
2) Viral detection: Throat swab specimens were collected immediately upon admission. Viral nucleic acids were extracted and amplified using commercial kits following the manufacturers' protocols, with subsequent multiplex PCR analysis conducted on the Dx 3500 genetic analyzer.

### Clinical hematological parameters included in the observational study

At admission, 2 mL venous blood was collected from the antecubital vein into EDTA-anticoagulated tubes. Specimens underwent comprehensive analysis for C-Reactive Protein (CRP), Serum Amyloid A (SAA), and Complete Blood Count (CBC), including White Blood Cell count (WBC), Neutrophil count (Neu), Lymphocyte count (Lym), and Monocyte count (Mon). Neutrophil-to-Lymphocyte Ratio (NLR) and Monocyte-to-Lymphocyte Ratio (MLR) were calculated from CBC data.^4^, ^5^, ^6^ All analyzed parameters are systematically documented in Table S2.

### Statistical data analysis

Statistical analyses were performed using SPSS 26.0 software, with figures generated using GraphPad Prism 8. Normally distributed continuous data are presented as mean ± Standard Deviation (x̄ ± s), while non-normally distributed data are expressed as median with interquartile range [M (Q1, Q3)]. Between-group comparisons for normally distributed data utilized the independent samples *t*-test; non-normally distributed data were compared using the Mann-Whitney *U* test. Categorical variables were analyzed using the Chi-Square test or Fisher's exact test, as appropriate.

Receiver Operating Characteristic (ROC) curves were generated in SPSS 26.0 to evaluate model discrimination, quantified by the Area Under the Curve (AUC). The optimal cut-off value was determined by maximizing the Youden index (sensitivity + specificity - 1) within the study cohort to optimize diagnostic accuracy (not pre-specified to avoid bias). Statistical significance was established at *p* < 0.05 for all analyses.

## Results

### Estimation of sample size

Sample size estimation was performed using GPower 3.1 (α = 0.05, β = 0.2, effect size *d* = 0.5). The minimum sample size required to achieve 80 % statistical power is 148. The final cohort size was 173, which was sufficient to detect the differences in AUC and biomarker parameters between the respiratory tract infection groups.

### Pathogen distribution in patients with respiratory tract infection

**Fig. 2:**
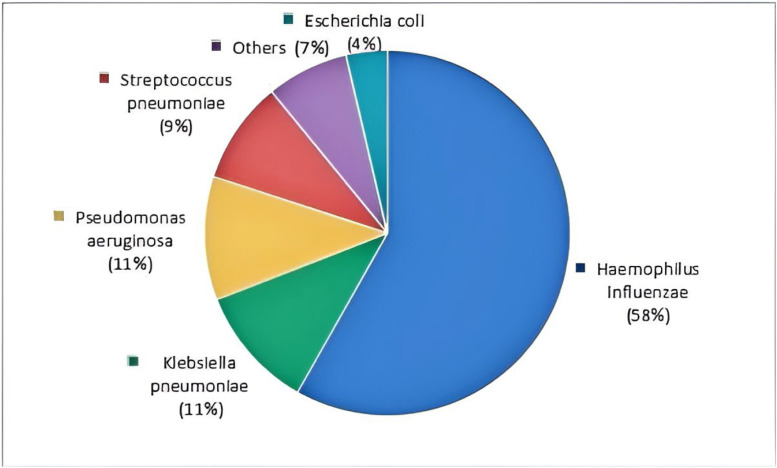
Percentage of bacterial subtypes in respiratory tract infectious patients.

Analysis of bacterial isolates from patients with respiratory tract infections revealed Gram-negative bacilli as the predominant pathogens. Haemophilus influenzae and Klebsiella pneumoniae were the most frequently identified species within this group. Gram-positive bacteria accounted for a secondary proportion of pathogens, primarily represented by Streptococcus pneumoniae (Table 1; Fig. 2). Among viral pathogens, influenza A virus and SARS-CoV-2 were the predominant respiratory viruses associated with infections.

**Table 1:** Types of bacteria from respiratory tract infectious patients.

Bacterial species	Bacterial type	Number	Constituent ratio
Haemophilus influenzae	Gram-negative bacteria	32	58 %
Klebsiella pneumoniae	Gram-negative bacteria	6	11 %
Pseudomonas aeruginosa	Gram-negative bacteria	6	11 %
Streptococcus pneumoniae	Gram-positive bacteria	5	9 %
*Escherichia coli*	Gram-negative bacteria	2	4 %
Others		4	7 %
Total		55	100 %

### Hematological parameter profiles in the study cohort

**Fig. 3:**
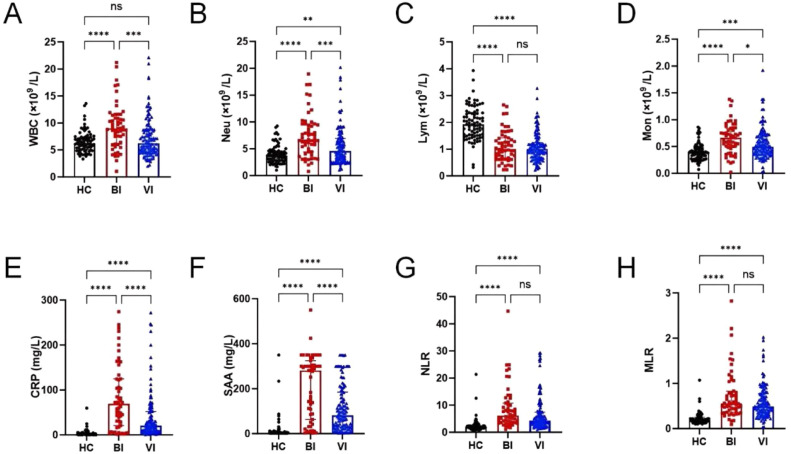
The difference of CRP, SAA, and blood routine parameters among groups Data presented as the median and interquartile range. (A‒H) The difference of WBC, Neu, Lym, Mon, CRP, SAA, NLR and MLR in three groups, respectively. HC, Healthy Control; BI, Bacterial Infection; VI, Viral Infection; “ns”, No significance (*p* > 0.05); * *p* < 0.05; ** *p* < 0.01; *** *p* < 0.001; **** *p* < 0.0001.

Hematological parameter profiles across the study groups are detailed in Table 2 and Fig. 3. Compared with the viral infection group and the healthy control group, the median levels of WBC, Neu, Mon, CRP, SAA, NLR, and MLR in the bacterial infection group were higher than those in the viral infection group and the healthy control group. Especially, the median levels of CRP (68.89 mg/L) and SAA (280.75 mg/L) in the bacterial infection group were significantly higher than those of CRP (20.26 mg/L) and SAA (81.16 mg/L) in the viral infection group and the healthy control group (CRP = 1.67 mg/L; SAA = 2.96 mg/L; *p* < 0.05). At the same time, compared with the healthy control group, the Neu, Mon, CRP, and SAA in the viral infection group showed moderate increases. In contrast, Lym levels were significantly decreased in both bacterial and viral infection groups compared to healthy controls.

**Table 2:** Characteristics of hematological parameters of the enrolled participants.

Parameter	Bacterial infection group	Viral infection group	Healthy control group
WBC (× 10^9^/L)	8.96 (6.25, 11.17)	6.22 (4.64, 8.73)	6.20 (5.09, 7.34)
Neu (× 10^9^/L)	6.69 (4.43, 9.54)	4.55 (2.97, 6.92)	3.72 (2.75, 4.59)
Lym (× 10^9^/L)	1.00 (0.70, 1.41)	1.00 (0.71, 1.23)	1.93 (1.52, 2.36)
Mon (× 10^9^/L)	0.66 (0.49, 0.82)	0.49 (0.35, 0.68)	0.39 (0.29, 0.49)
CRP (mg/L)	68.89 (24.86, 124.39)	20.26 (8.26, 51.76)	1.67 (0.66, 4.36)
SAA (mg/L)	280.75 (73.89, 314.92)	81.16 (29.10, 182.91)	2.96 (2.00, 8.08)
NLR	6.13 (3.85, 10.42)	4.29 (2.89, 7.37)	1.80 (1.38, 2.51)
MLR	0.55 (0.38, 0.82)	0.49 (0.35, 0.77)	0.20 (0.16, 0.26)

### Diagnostic utility of hematological parameters in respiratory tract infections

**Fig. 4:**
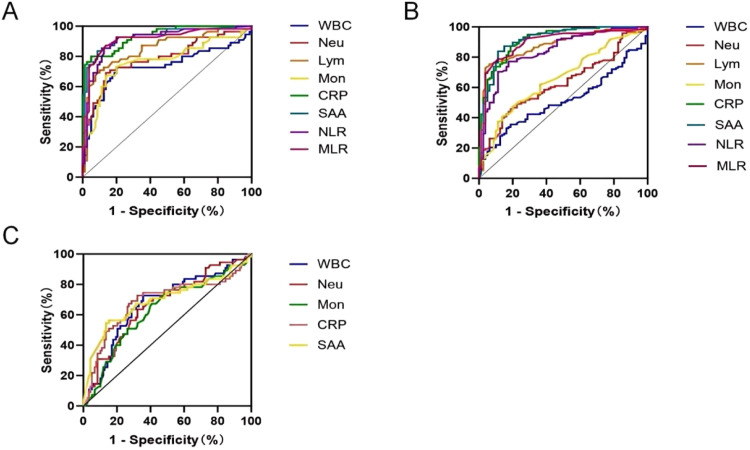
ROC curve of the diagnostic models. (A) The ROC curve of the diagnostic model established between the bacterial respiratory tract infection group and healthy control group. (B) The ROC curve of the diagnostic model established between the viral respiratory tract infectious group and healthy control group. (C) The ROC curve established by the blood parameters for differentiating bacterial from viral respiratory tract infection.

To establish a diagnostic model, hematological parameters were analyzed and compared between the healthy control group and the RTIs group. Receiver Operating Characteristic (ROC) curve analysis assessed the diagnostic efficacy of these parameters. As shown in Fig. 4A‒B and Table 3, the WBC demonstrated limited diagnostic value (low AUC) for viral RTIs, although it retained clinical significance for bacterial infections. In contrast, other parameters (including Neu, Mon, NLR, MLR, CRP and SAA) all demonstrated relatively good diagnostic performance in both models. Notably, most parameters showed superior diagnostic utility for bacterial infections compared to viral infections. In addition, Lym was a clear exception, whose diagnostic accuracy (AUC) was marginally higher in the viral infection model than in the bacterial infection model.

**Table 3:** Diagnostic efficacy of hematological parameters for respiratory tract infections.

**Parameters**	**Bacterial infection group vs. Healthy control group**		**Viral infection group vs. Healthy control group**	
	**AUC (95 % CI)**	**p*-*value**	**AUC (95 % CI)**	**p*-*value**
WBC	0.733 (0.636-0.830)	<0.001	0.514 (0.434-0.594)	0.746
Neu	0.789 (0.706-0.873)	<0.001	0.627 (0.550-0.704)	0.002
Lym	0.855 (0.786-0.923)	<0.001	0.876 (0.825-0.927)	<0.001
Mon	0.760 (0.671-0.849)	<0.001	0.661 (0.586-0.737)	0.001
CRP	0.935 (0.896-0.975)	<0.001	0.915 (0.876-0.954)	<0.001
SAA	0.928 (0.880-0.975)	<0.001	0.913 (0.869-0.956)	<0.001
NLR	0.911 (0.858-0.964)	<0.001	0.841 (0.785-0.897)	<0.001
MLR	0.900 (0.841-0.960)	<0.001	0.896 (0.849-0.943)	<0.001

Subsequently, the authors developed a diagnostic model to differentiate bacterial from viral infections using hematological parameters, which showed statistically significant differences between the infection groups (Fig. 3). With the viral infection group as the reference, the model demonstrated significant discriminative capacity between the two etiologies.

ROC curve analysis evaluated the model's diagnostic performance. Among single parameters, SAA exhibited the highest discriminatory ability (AUC = 0.693), followed by CRP (AUC = 0.686) (Fig. 4C). Moreover, the details of each parameter in distinguishing bacterial from viral respiratory infections are presented in Table 4.

**Table 4:** Efficacy of single hematological parameter in differentiating bacterial from viral respiratory tract infections.

**Parameters**	**AUC (95 % CI)**	**Sensitivity**	**Specificity**	**Cut-off value**	**p-value**
WBC	0.673 (0.585-0.761)	0.727	0.644	7.48	<0.001
Neu	0.666 (0.578-0.753)	0.636	0.678	6.13	<0.001
Mon	0.630 (0.539-0.722)	0.673	0.593	0.54	0.006
CRP	0.686 (0.591-0.781)	0.727	0.678	30.41	<0.001
SAA	0.693 (0.599-0.788)	0.564	0.847	250.47	<0.001

### Development and diagnostic performance of combined parameters

As shown in Table 4, individual hematological parameters demonstrated suboptimal performance in discriminating bacterial from viral RTIs. To address this limitation, the authors integrated the five parameters through algorithmic feature selection and multivariate logistic regression, generating combined diagnostic indices (Table 5). ROC curve analysis identified the third parameter combination as optimal, exhibiting superior diagnostic efficacy (AUC = 0.764) compared to both individual parameters and alternative combinations. Notably, diagnostic performance was not necessarily proportional to parameter count: Combination 5 (incorporating all five variables) achieved a lower AUC (0.755) than Combination 3, indicating diminishing returns with excessive variable inclusion.

**Table 5:** Efficacy of combined parameters in differentiating bacterial from viral respiratory tract infections.

**Combined parameters**	**AUC (95 % CI)**	**p-value**
① WBC + Neu + Mon	0.686 (0.599-0.773)	<0.001
② CRP + SAA	0.695 (0.601-0.789)	<0.001
③ WBC + Neu + Mon + CRP	0.764 (0.692-0.835)	<0.001
④ WBC + Neu + Mon + SAA	0.748 (0.671-0.824)	<0.001
⑤ WBC + Neu + Mon + CRP + SAA	0.755 (0.681-0.829)	<0.001

Methodological evaluation of the optimal parameter combination demonstrated a sensitivity of 75.2 % (95 % CI: 0.681ཞ0.813) and specificity of 89.3 % (95 % CI: 0.835ཞ0.932). This combined index significantly outperformed all individual parameters in diagnostic accuracy. Its balanced sensitivity-specificity profile highlights superior clinical utility for discriminating bacterial versus viral respiratory infections. These findings indicate that strategic parameter integration enhances diagnostic reliability more effectively than single-marker approaches. The combined model represents a clinically practical advancement over conventional methods, demonstrating tangible improvements in real-world diagnostic utility.

## Discussion

Blood testing represents a pivotal diagnostic modality for differentiating respiratory infections, utilizing routinely measured parameters including complete blood count indices (e.g., leukocyte subsets, NLR) and inflammatory biomarkers such as CRP and SAA. These assays provide rapid turnaround times, operational convenience, and high reproducibility, making them indispensable for initial clinical assessment. However, individual parameters demonstrate limited diagnostic specificity in distinguishing bacterial from viral etiologies. This limitation becomes particularly critical when etiological diagnostic testing (Which is frequently delayed by prolonged processing times, suboptimal sampling techniques, contamination risks, or low pathogen detection rates) fails to deliver timely guidance. Integrating multiple variables significantly enhances diagnostic accuracy, thereby facilitating targeted clinical interventions. Such combined models bridge diagnostic gaps during the pre-analytical phase of etiological testing while enabling early pathogen classification. This approach optimizes antimicrobial stewardship and reduces reliance on empirical treatment.

This study revealed significantly elevated levels of Complete Blood Count (CBC) parameters, particularly WBC and Neu, along with inflammatory biomarkers (CRP, SAA) in bacterial RTIs compared to viral infections and healthy controls. This pattern aligns with known pathophysiological responses to gram-negative bacterial endotoxins (e.g., lipopolysaccharides), which trigger interleukin-mediated leukocyte chemotaxis and acute-phase protein synthesis.^7^^,^^8^ As quantified in Table 2, bacterial infections demonstrated pronounced neutrophilia, with Neu levels exceeding both viral infections and established reference ranges. This reinforces neutrophil count's utility as a preliminary bacterial screening biomarker. Elevated Neu values strongly correlate with systemic inflammatory burden, serving as a key discriminator for bacterial versus viral etiology.^9^, ^10^, ^11^, ^12^

Contrary to typical viral infection profiles, both bacterial and viral groups exhibited reduced absolute Lym relative to healthy controls. This paradoxical finding may reflect compartmentalized immune responses. In viral infections, lymphocyte sequestration to pulmonary tissues (primary sites of viral replication and clearance) may reduce peripheral Lym. In bacterial infections, neutrophilia-induced relative lymphopenia (due to proportional shifts in leukocyte populations) likely explains the observed decrease, which is distinct from virally mediated depletion mechanisms.^13^, ^14^, ^15^ Notably, certain viruses may directly suppress lymphocyte populations through immune dysregulation.^16^, ^17^, ^18^

Bacterial infections typically provoke more pronounced tissue damage and systemic inflammation than viral etiologies, necessitating vigilant monitoring of inflammatory biomarkers. CRP and SAA serve as critical indicators in this context. While CRP levels > 140 mg/L strongly predict bacterial co-infection or primary bacterial etiology,^19^ its behavior in early viral infections is nuanced: modest CRP elevation (typically < 50 mg/L) may indicate subclinical inflammation despite absent severe symptoms. CRP's diagnostic advantage lies in its differential elevation patterns: 1) Significantly greater increases in bacterial infections, 2) Minimal-to-absent elevation in most viral cases (Table 2). SAA offers complementary diagnostic value. Though elevated in both infection types, bacterial infections demonstrate disproportionately higher SAA levels (median 280.75 vs. 81.16 mg/L; Table 2). Notably, SAA exhibits superior sensitivity for low-grade inflammation, detecting early viral infections even when CRP remains normal. This capacity to identify subtle immune activation makes SAA particularly valuable for triaging ambiguous presentations, addressing CRP's delayed response in viral contexts.^20^, ^21^, ^22^

The present study indicated that the diagnostic efficacy of a single parameter is limited (the AUC of SAA is highest at only 0.693, which is <0.7). Moreover, as shown in Table 4, routine blood parameters demonstrate relatively low efficacy. Inflammatory markers (SAA and CRP) show slightly better efficacy than routine parameters, while their combination significantly improves diagnostic performance (Table 5), confirming the necessity of composite parameters. Integrating multiple individual parameters enhances diagnostic efficacy. However, comparison of combination parameters (3) and (5) in Table 5 reveals that adding more variables does not proportionally enhance performance. This indicates that composite models should prioritize variable quality over quantity. Therefore, the authors propose a tiered diagnostic approach for RTIs: Conduct preliminary screening using Complete Blood Count (CBC) and CRP first, then perform SAA testing for indeterminate cases. This sequential strategy may reduce unnecessary testing, potentially alleviating patients' economic burden. Prospective evaluation remains necessary to assess impacts on prescribing behavior.

Additionally, since age influences immune responses, the significant difference between healthy controls (40.8 ± 13.7 years) and infection groups (∼70-years; Table S1) may introduce bias. Age-adjusted logistic regression was attempted, but limited subgroup sizes constrained interpretability. Future studies should recruit age-matched controls or implement statistical age-adjustment. Furthermore, prospective validation in comorbid populations (e.g., diabetes, COPD) is essential to assess real-world utility, as exclusion criteria may overestimate model performance in healthier cohorts.

In conclusion, individual parameters show limited utility, while multi-parameter combinations moderately improve performance. However, composite models do not benefit indiscriminately from added variables ‒ quality outweighs quantity. For viral respiratory infections, SAA combined with routine blood parameters serves as an effective preliminary screening tool, whereas CRP demonstrates higher diagnostic value for bacterial infections. Calculated indices like NLR (reflecting inflammation severity) provide supplementary value.^23^^,^^24^

This study focuses on widely available clinical blood parameters. Although individual parameters show moderate efficacy and the model is relatively simple, their combination achieves clinically useful performance. The authors established optimal cut-off values for individual parameters, providing practical reference for primary care settings. Compared to pathogen detection, this model is more economical, user-friendly, and workflow-compatible.

While this parameter combination may help differentiate bacterial/viral respiratory infections early,^25^, ^26^, ^27^ prospective validation remains necessary to evaluate impacts on clinical decision-making, antibiotic stewardship, and patient outcomes. The modest sample size (*n* = 173) under strict inclusion criteria may limit generalizability. A major limitation is the absence of internal validation (e.g., k-fold cross-validation or bootstrapping), increasing overfitting risk. Therefore, the reported performance metrics (Table 4) may represent optimistic estimates. Generalizability to clinical practice requires cautious interpretation and rigorous external validation.

## Funding

The authors acknowledge the financial support from the 10.13039/501100004300Scientific Research Foundation for Advanced Talents, Affiliated Hospital of North Sichuan Medical College (Grant No.: 2023GC009).

## Data availability statement

Data supporting the findings of this study are available upon request from the corresponding author. This data is not made public due to privacy or ethical restrictions.

## Ethical statement

This study was approved by the Ethics Committee of Chongqing People's Hospital (Approval No.: KY S2022-054-01). Throughout the entire research process, the authors strictly adhered to the requirements and suggestions put forward by the committee to ensure the scientific nature, legality, and ethics of the research. Due to the retrospective nature of the study, written informed consent was waived, but all patient data were anonymized and handled in accordance with the Declaration of Helsinki.

## CRediT authorship contribution statement

**Yu Zhou:** Conceptualization, Resources, Writing – original draft. **Lei Xu:** Writing – original draft. **Xiaowu Zhong:** Writing – original draft. **Xiaolan Guo:** Writing – review & editing. **Qiang Ma:** Conceptualization, Writing – review & editing.

## Conflict of Interest

The authors declare no conflicts of interest.

## Appendix Supplementary materials

Image, application 1
