# Supplementary material for: Differentiating bacterial from viral respiratory tract infections using CRP, SAA, and blood routine parameters: A retrospective cohort study
Source: Clinics (Sao Paulo). 2025 Nov 26;80:100845. doi: 10.1016/j.clinsp.2025.100845 (PMC12702070; doi:10.1016/j.clinsp.2025.100845)
Supplement: Supplementary file 1 [file mmc1.docx]

**CLINICS-D-25-00572_Supplementary Material**

**Table S1** General clinical characteristics of study participants.

|  | **Bacterial infection group (%)** | **Virus infection group (%)** | **Healthy control (%)** |
| --- | --- | --- | --- |
| **Number** | 55 | 118 | 80 |
| **Age** | 70.8±14.3 | 68.2±15.7 | 40.8±13.7 |
| **Gender** |  |  |  |
| Male | 39 (70.9) | 64 (54.2) | 45 (56.3) |
| Female | 16 (29.1) | 54 (45.8) | 35 (43.7) |

**Table S2** Reference interval of hematological parameters of participants.

| **Hematological parameters** | **Reference interval** | **Units** | **Positive decision value** |
| --- | --- | --- | --- |
| WBC | (3.50‒9.50) ×10^9^ | pcs/L | >9.50×10^9^ |
| Neu | (1.80‒6.30) ×10^9^ | pcs/L | >6.30×10^9^ |
| Lym | (1.10‒3.20) ×10^9^ | pcs/L | <1.10×10^9^ |
| Mon | (0.10‒0.60) ×10^9^ | pcs/L | >0.60×10^9^ |
| CRP | 0.00‒10.00 | mg/L | >10.00 |
| SAA | 0.00‒10.00 | mg/L | >10.00 |
| NLR | 0.90‒3.82 | / | >3.82 |
| MLR | 0.09‒0.33 | / | >0.33 |

pcs/L, the number of cells counted per liter of blood; mg/L, A unit of the amount of protein per liter of blood.
